# A231 EFFECTIVENESS OF DUAL BIOLOGIC THERAPY IN PEDIATRIC INFLAMMATORY BOWEL DISEASE: A MULTICENTRE CANADIAN STUDY

**DOI:** 10.1093/jcag/gwad061.231

**Published:** 2024-02-14

**Authors:** F Diaz, R Belaghi, T Walters, E Chea, D Friedland, E Wine, C Deslandres, H Huynh, J deBruyn, D Mack, A Otley, A Ricciuto, E I Benchimol, M W Carroll, P Church, N Carman, A Shaikh, A Griffiths, W El-Matary

**Affiliations:** University of Manitoba Department of Pediatrics and Child Health, Winnipeg, MB, Canada; Children's Hospital of Eastern Ontario Research Institute, Ottawa, ON, Canada; SickKids Inflammatory Bowel Disease Centre, The Hospital for Sick Children, University of Toronto, Toronto, ON, Canada; SickKids Inflammatory Bowel Disease Centre, The Hospital for Sick Children, University of Toronto, Toronto, ON, Canada; SickKids Inflammatory Bowel Disease Centre, The Hospital for Sick Children, University of Toronto, Toronto, ON, Canada; Edmonton Pediatric IBD Clinic, Stollery Children’s Hospital, University of Alberta, Edmonton, AB, Canada; Universite de Montreal, Montreal, QC, Canada; Edmonton Pediatric IBD Clinic, Stollery Children’s Hospital, University of Alberta, Edmonton, AB, Canada; University of Calgary, Calgary, AB, Canada; Children's Hospital of Eastern Ontario Research Institute, Ottawa, ON, Canada; Dalhousie University, Halifax, NS, Canada; SickKids Inflammatory Bowel Disease Centre, The Hospital for Sick Children, University of Toronto, Toronto, ON, Canada; SickKids Inflammatory Bowel Disease Centre, The Hospital for Sick Children, University of Toronto, Toronto, ON, Canada; Edmonton Pediatric IBD Clinic, Stollery Children’s Hospital, University of Alberta, Edmonton, AB, Canada; SickKids Inflammatory Bowel Disease Centre, The Hospital for Sick Children, University of Toronto, Toronto, ON, Canada; SickKids Inflammatory Bowel Disease Centre, The Hospital for Sick Children, University of Toronto, Toronto, ON, Canada; SickKids Inflammatory Bowel Disease Centre, The Hospital for Sick Children, University of Toronto, Toronto, ON, Canada; SickKids Inflammatory Bowel Disease Centre, The Hospital for Sick Children, University of Toronto, Toronto, ON, Canada; University of Manitoba Department of Pediatrics and Child Health, Winnipeg, MB, Canada

## Abstract

**Background:**

The effectiveness of combining biologics for pediatric inflammatory bowel disease (IBD) is under-investigated.

**Aims:**

To evaluate the effectiveness of dual biologic therapy in children with IBD.

**Methods:**

Children and adolescents ampersand:003C 17 years old with IBD enrolled in the Canadian Children IBD Network (CIDsCANN) who received any biologic therapy were included. Patients were classified as cases if they received two biologics simultaneously, and controls if they were switched from one biologic to another. All cases and controls who met inclusion criteria were analyzed.

Baseline demographic and disease-specific data were collected. The primary outcome was clinical activity indices at the end of the 1-year period following the start of dual biologic therapy or the switch to a different biologic.

The Wilcoxon signed rank test (for continuous variables) and the Fisher exact test (for nominal variables) were used to compare demographic data. A multivariable Cox proportional hazard model was used to infer about disease activity reduction between the two groups. p ampersand:003C 0.05 was used to determine statistical significance.

**Results:**

Twenty-six cases and 194 controls fulfilled the inclusion criteria. Demographic and disease-specific data are summarized in Table 1.

The most common combination was ustekinumab with vedolizumab in 11 (42.3%) patients; while the most common first biologic in the control group was infliximab (104, 54.1%) and the second was ustekinumab (109, 56.2%). The most common indication for the biologic therapy addition or switch was unsatisfactory clinical response.

No differences between cases and controls in reductions of disease activity indices were seen at the end of the 1-year period (Figure 1).

**Conclusions:**

In children with IBD, dual biologic therapy was not more effective than switching from one biologic to another. We are currently investigating the safety of this approach.

Baseline data

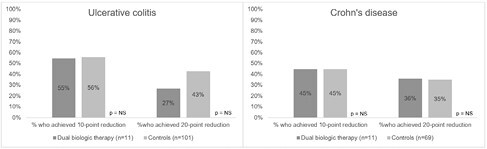

Proportion of patients with 10- and 20-point reduction in clinical activity indices at the end of the 1-year period

**Funding Agencies:**

None

**Table:** 

	Dual biologic (n=26)	Control (n=194)
***Sex***		
Male	18 (69%)	103 (53%)
Female	8 (31%)	91 (47%)
***Age at diagnosis (years)***		
Mean (SD)	10.9 (3.11)	11.5 (3.46)
***Diagnosis***		
Ulcerative colitis	12 (46%)	107 (55%)
Crohn's disease	14 (54%)	87 (45%)
***Disease duration (years)***		
Mean (SD)	4.47 (2.43)	4.34 (2.31)
***Baseline disease activity index***		
PUCAI mean (SD)	25.0 (21.3)	25.3 (23.8)
PCDAI mean (SD)	29.0 (22.2)	29.3 (23.0)

